# 

**Published:** 1870-03

**Authors:** 


### CONTENTS.

ORIGINAL COMMUNICATIONS.

Facts *vs.* Theories ........ 109
Address at the Twenty-Fourth Annual Commencement of the Ohio College of Dental Surgery ........ 119
Reply on behalf of the Class ........ 125
Aluminum as a Base for Artificial Teeth ........ 128
Safety from Exploding Vulcanizers ........ 131

PROCEEDINGS OF SOCIETIES.

Proceedings of the Michigan State Dental Association ........ 132
The Missouri Valley Dental Society ........ 144

EDITORIAL.

The Dental Profession ........ 145
New Bases for Artificial Teeth ........ 147
Heavy Foil ........ 148
The Mississippi Valley Dental Society ........ 149
The Law of "Vis Viva" ........ 150

